# Innate lymphoid cells are reduced in pregnant HIV positive women and are associated with preterm birth

**DOI:** 10.1038/s41598-020-69966-0

**Published:** 2020-08-06

**Authors:** Charlene Akoto, Christina Y. S. Chan, Chrystelle O. O. Tshivuila-Matala, Krithi Ravi, Wei Zhang, Manu Vatish, Shane A. Norris, Joris Hemelaar

**Affiliations:** 1 Nuffield Department of Women’s & Reproductive Health, University of Oxford, The Women’s Centre, John Radcliffe Hospital, Oxford, OX3 9DU UK; 2 South African Medical Research Council Developmental Pathways for Health Research Unit, Department of Paediatrics, School of Clinical Medicine, University of the Witwatersrand, Johannesburg, South Africa

**Keywords:** Immunology, Biomarkers, Pathogenesis

## Abstract

Preterm birth is the leading cause of neonatal and child mortality worldwide. Globally, 1.4 million pregnant women are estimated to be living with HIV/AIDS, the majority of whom live in sub-Saharan Africa. Maternal HIV infection and antiretroviral treatment (ART) have been associated with increased rates of preterm birth, but the underlying mechanisms remain unknown. Acute HIV infection is associated with a rapid depletion of all three subsets of innate lymphoid cells (ILCs), ILC1s, ILC2s and ILC3s, which is not reversed by ART. ILCs have been found at the maternal–fetal interface and we therefore investigated the potential association between maternal HIV infection, peripheral ILC frequencies and preterm birth. In our study of pregnant South African women with accurately dated pregnancies, we show that maternal HIV infection is associated with reduced levels of all three ILC subsets. Preterm birth was also associated with lower levels of all three ILC subsets in early pregnancy. ILC frequencies were lowest in HIV positive women who experienced preterm birth. Moreover, ILC levels were reduced in pregnancies resulting in spontaneous onset of preterm labour and in extreme preterm birth (< 28 weeks gestation). Our findings suggest that reduced ILC frequencies may be a link between maternal HIV infection and preterm birth. In addition, ILC frequencies in early pregnancy may serve as predictive biomarkers for women who are at risk of delivering preterm.

## Introduction

Globally, preterm birth (PTB) is the leading cause of neonatal and child mortality, accounting for approximately 18% of deaths in 2016^1^. In those infants who survive, PTB is associated with an increased risk of short- and long-term morbidities^2^. Preterm birth is a syndrome caused by multiple pathological processes and the underlying mechanisms remain elusive, holding back progress in prediction, prevention and treatment^3^.

Of the 36.9 million people estimated to be living with HIV/AIDS worldwide^4^, approximately 1.4 million are pregnant women, the majority of whom reside in sub-Saharan Africa^5^. A systematic review and meta-analysis conducted by our group revealed that HIV positive expectant mothers not receiving antiretroviral therapy (ART) experienced higher rates of PTB, low birth weight, small-for-gestational-age, and stillbirth than HIV negative women^6^. While ART administered during pregnancy is effective at reducing maternal morbidity and mortality as well as mother-to-child HIV transmission, ART does not reverse the effect of HIV on perinatal outcomes and may even exacerbate it, although reports are conflicting^7–14^.

HIV infection is characterised by a progressive depletion of CD4+ T cells and persistent immune activation^15^. In addition, it was recently reported that innate lymphoid cells (ILCs) are depleted during acute HIV infection^16^. ILCs are immune effectors which function to provide protective responses against pathogens and tumours and are also involved in lymphoid organogenesis during fetal development^17^. They can be divided into three groups, ILC1s, ILC2s and ILC3s, based on surface marker expression, cytokine secretion profiles and transcriptional regulation, and are characterised by their lack of expression of antigen specific receptors and known immune cell lineage markers^18^. In response to stress signals, microbial compounds and the local cytokine milieu, ILC1s, ILC2s and ILC3s secrete a range of effector cytokines, which mirror those of CD4 T helper (Th) 1, Th2 and Th17 cells, respectively. ILCs are enriched in tissues, particularly at mucosal surfaces e.g. of the intestines, lungs, uterus and skin but are also found in lower frequencies in the peripheral blood^17^. Their location at barrier surfaces aids their role as early responders during an immune response, however, they are also involved in a number of immunopathologies^19^.

A number of studies in HIV infected patients have reported decreased or modulated ILC frequencies, which may be compartment specific^16,20–22^. Depletion of all three ILC subsets was observed in the blood of HIV infected patients not receiving ART, coinciding with peak viremia and, unlike CD4 T cell counts, persisted into chronic infection even after the resolution of acute viremia^16^. In those patients on effective ART (suppression of viremia, recovery of CD4 T cell counts), ILC1 and ILC2 frequencies failed to recover, and ILC3s were only partially reconstituted even after 2 years of successful ART. Only when ART was initiated within 14 days of HIV transmission were all three ILC subsets preserved^16^. Others report that in the peripheral blood total ILC frequencies, as well as ILC3s, are lower in HIV infected patients, with lower frequencies negatively correlating with viremia and associated with increased disease severity^23^. Furthermore, cells secreting the ILC3 associated cytokines IL-17 and IL-22 are depleted from the colon mucosa and ILC1s and ILC3s are lost from the ileum and colon of HIV infected patients^21,23,24^.

As well as the peripheral blood and gut, ILCs are located in the uterus and the decidua^25–30^, suggesting a role in pregnancy. All three ILC subsets have been identified in the human uterus outside of pregnancy^25,26^ and have been found in the uterus and decidua during human as well as murine pregnancy^25,27,28,31,32^. While similar ILC levels have been found in the non-pregnant endometrium and the first trimester decidua^25,26^, the use of mouse models has allowed the tracking of ILC frequencies during pregnancy and shown that uterine ILC1, ILC2 and ILC3 levels increase throughout gestation^26,31,33^. In addition, ILCs positive for IFN-γ, IL-5, or IL-13 increase during early and mid-gestation^25,31^ and suggest ILCs may be involved in the processes of implantation and immune tolerance via the release of pro-inflammatory and Th2 cytokines respectively. The mechanisms influencing adverse pregnancy outcomes are not fully understood but changes in uterine and peripheral ILC frequencies, and their accompanying cytokines, have been associated with adverse pregnancy outcomes including spontaneous preterm labour and murine pregnancy loss^28,34,35^. We therefore investigated the potential association between HIV infection in human pregnancy, peripheral ILC frequencies, and adverse pregnancy outcomes, specifically preterm birth.

## Results

### Patient characteristics

Forty-six HIV positive (HIV+) and 45 HIV negative (HIV−) women were largely comparable at baseline (Table 1). The maternal age of HIV+ women was higher than that of HIV− women (*p* = 0.031) with fewer years of education (*p* = 0.005). Importantly, there were no other significant differences, either in obstetric history, parity, pre-pregnancy BMI, smoking status or alcohol intake (Table 1).

**Table 1:** Patient characteristics.

	HIV+ patients	HIV− patients	Statistical comparison
Number of patients	46	45	
Maternal age (median [IQR])	33 [28–37]	29 [26–33]	*p* = 0.031
Pre-pregnancy body mass index (mean [SD])	27.6 [4.3]	26.2 [3.5]	*p* = 0.106
Number of previous pregnancies (median [IQR])	2 [1–3]	2 [1–3]	*p* = 0.903
History of adverse pregnancy outcome (number [%])	25 [54%]	28 [62%]	*p* = 0.275
Smoking during pregnancy (number [%])			
Yes	5 [11%]	2 [4%]	*p* = 0.435
No	41 [89%]	43 [96%]	
Alcohol intake during pregnancy (number [%])			
Yes	8 [17%]	4 [9%]	*p* = 0.354
No	38 [83%]	41 [91%]	
Number of years of education (median [IQR])	12 [11–12]	12 [12–12]	*p* = 0.005
Antiretroviral therapy initiation category (number [%])			
Preconception	17 [37%]	N/A	
Post-conception	18 [39%]	N/A	
Unknown	11 [24%]	N/A	
Number of samples			
Trimester 1	25	25	
Trimester 2	36	32	
Trimester 3	14	16	
Weeks + days of gestation at sample collection (median [range])			
Trimester 1	12 + 4 [8 + 0 − 13 + 6]	12 + 0 [8 + 0 − 14 + 2]	*p* = 0.573
Trimester 2	26 + 1 [23 + 4 − 28 + 1]	26 + 0 [20 + 6 − 27 + 6]	*p* = 0.444
Trimester 3	35 + 3 [31 + 1 − 37 + 4]	35 + 4 [30 + 2 − 39 + 0]	*p* = 0.997
Preterm birth (PTB) (number [%])	22 [48%]	25 [56%]	*p* = 0.461
Moderate-to-late PTB	13 [28%]	10 [22%]	*p* = 0.508
Very PTB	3 [7%]	6 [13%]	*p* = 0.316
Extreme PTB	6 [13%]	9 [20%]	*p* = 0.371

Characteristics of HIV positive and HIV negative pregnant women were compared using the appropriate statistical tests (Mann–Whitney-U test or unpaired t test for continuous variables; Fisher’s exact test or Chi squared test for categorical variables). History of adverse pregnancy outcome: at least one occurrence of preterm birth, low birth weight, miscarriage, stillbirth or neonatal death.

### ILC frequencies are stable during pregnancy

**Figure 1:**
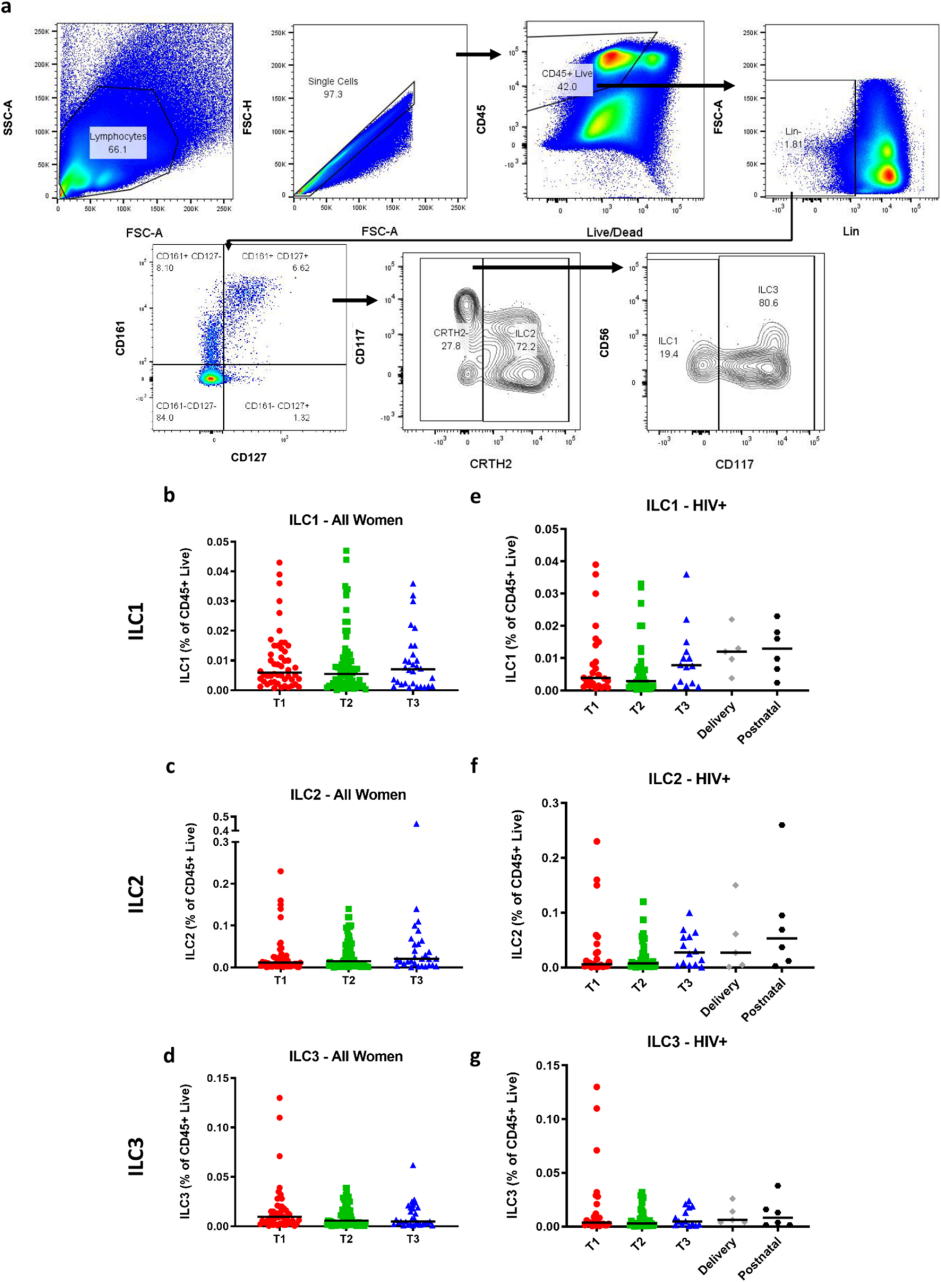
ILC1, ILC2 and ILC3 cells throughout pregnancy. (**a**) Gating strategy identifying ILC1, ILC2 and ILC3 cells among live, lineage negative (Lin-) CD45+ lymphocytes. (**b**) ILC1, (**c**) ILC2 and (**d**) ILC3 cell frequencies of all women studied (i.e. both HIV+ and HIV− women) during the first (T1), second (T2) and third (T3) trimester. (**e**) ILC1, (**f**) ILC2 and (**g**) ILC3 cell frequencies of HIV positive (HIV+) women during the first, second and third trimester and at delivery and six weeks postnatal. Data bars at median.

Using flow cytometry, we applied a gating strategy that identified three phenotypically distinct ILC populations in peripheral blood samples: Lin- CD45+ CD127+ CD161+ CRTH2− CD117− (ILC1), Lin- CD45+ CD127+ CD161+ CRTH2+ (ILC2) and Lin- CD45+ CD127+ CD161+ CRTH2− CD117+ (ILC3) (Fig. 1a). We analysed ILC frequencies (expressed as a percentage of CD45+ lymphocytes) in each trimester and found no change in the frequency of ILC1, ILC2 or ILC3 subsets over the course of pregnancy in all women (i.e. both HIV+ and HIV− women), or in HIV+ and HIV− women separately (Fig. 1b–g, Supplementary Fig. S1). Furthermore, in HIV+ women, for some of whom we had samples at delivery and 6 weeks postnatally, there were no significant differences between ILC frequencies during pregnancy and delivery or postnatal periods (Fig. 1e–g).

### ILC frequencies are lower in HIV positive women

**Figure 2:**
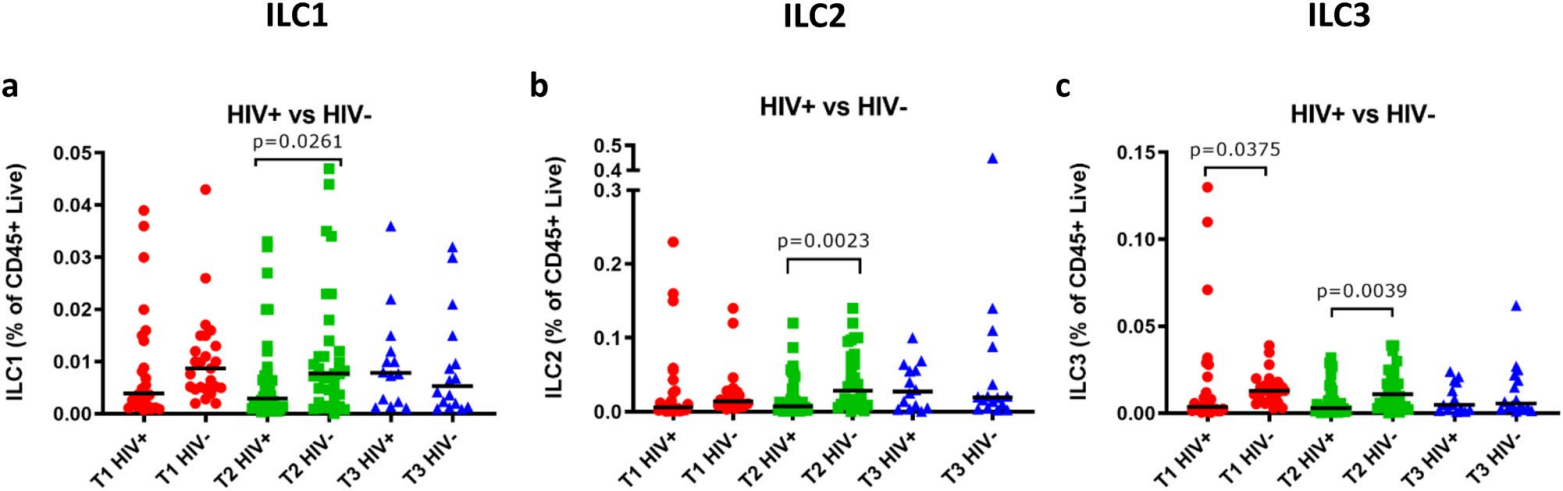
ILC1, ILC2 and ILC3 cells of HIV positive and HIV negative women throughout pregnancy. (**a**) ILC1, (**b**) ILC2 and (**c**) ILC3 cell frequencies of HIV positive (HIV+) and HIV negative (HIV−) women during the first (T1), second (T2) and third (T3) trimester. Data bars at median.

We compared ILC frequencies of HIV+ and HIV− women over the course of pregnancy. HIV+ women showed a significant reduction in the frequency of all three ILC subsets during the second trimester compared to HIV− women (Fig. 2a–c). Reductions in the ILC subsets of HIV+ women were also seen in the first trimester, but only reached statistical significance for ILC3s (Fig. 2c).

### ILC frequencies are not affected by the timing of antiretroviral therapy initiation

Reports have suggested a link between the timing of ART initiation and the risk of adverse pregnancy outcome, particularly preterm birth^7^. Therefore, we compared ILC frequencies of HIV− women and HIV+ women who initiated ART either pre- or post-conception. There were no differences in ILC frequencies between HIV+ women who initiated ART preconception or post-conception (Supplementary Fig. S2). However, consistent with previous results comparing HIV+ and HIV− women, frequencies of all three ILC subsets were lower in the second trimester in HIV+ women receiving either preconception or post-conception ART, compared to HIV− women (Supplementary Fig. S2).

### ILCs are lower in mothers who deliver preterm and lowest in HIV positive mothers who deliver preterm

**Figure 3:**
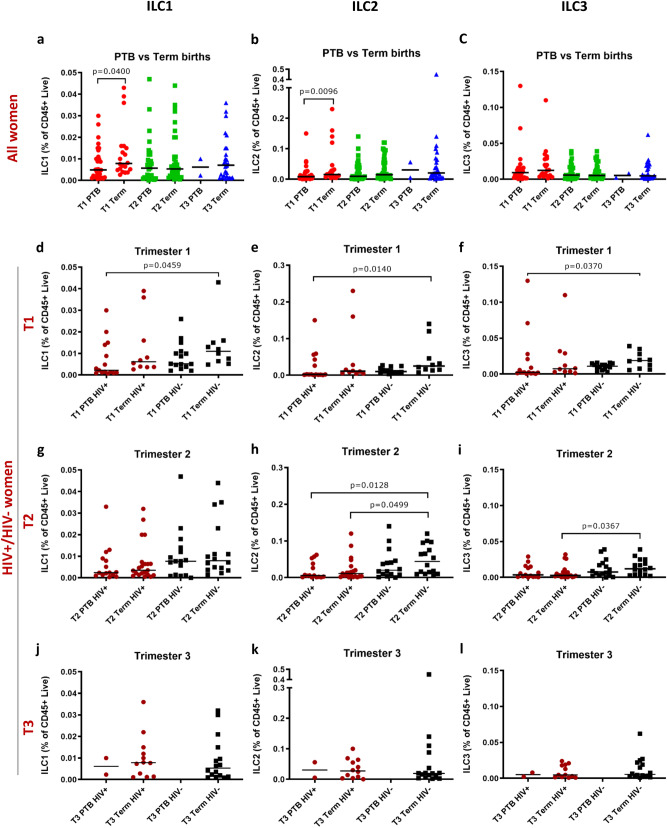
ILC1, ILC2 and ILC3 cells of HIV positive and/or HIV negative women with preterm or term births. (**a**) ILC1, (**b**) ILC2 and (**c**) ILC3 cell frequencies of women who delivered preterm (PTB) compared to those who delivered at term. (**d**–**f**) First (T1), (**g**–**i**) second (T2) and (**j**–**l**) third (T3) trimester ILC1, ILC2 and ILC3 frequencies, respectively, of HIV positive (HIV+) and HIV negative (HIV−) women who delivered preterm (PTB) or at term. Data bars at median.

PTB is the leading cause of neonatal and child mortality worldwide; therefore, we investigated ILC frequencies according to PTB status. In the first trimester, ILC1 and ILC2 frequencies were significantly lower in women who experienced PTB compared to those who delivered at term (Fig. 3a, b). A trend towards lower first trimester ILC3 frequencies associated with PTB was also apparent (Fig. 3c).

When PTB was analysed according to HIV status, first and second trimester ILC frequencies showed a stepwise decrease in all three ILC subsets (Fig. 3d–i): the highest frequencies were found in HIV− women who delivered at term, followed by HIV− women with PTB and HIV+ women with term births, and the lowest ILC frequencies were found in HIV+ women who delivered preterm (Fig. 3d–i). As a result, there were significant reductions in the frequencies of ILC1, ILC2 and ILC3 subsets in the first trimester in HIV+ women with PTB compared to HIV− women with term births, and of ILC2 cells in the second trimester (Fig. 3d–i). Too few data points were available for a comparable statistical analysis in the third trimester (Fig. 3j–l).

### ILC2s are lower in mothers with extreme preterm births compared to term

**Figure 4:**
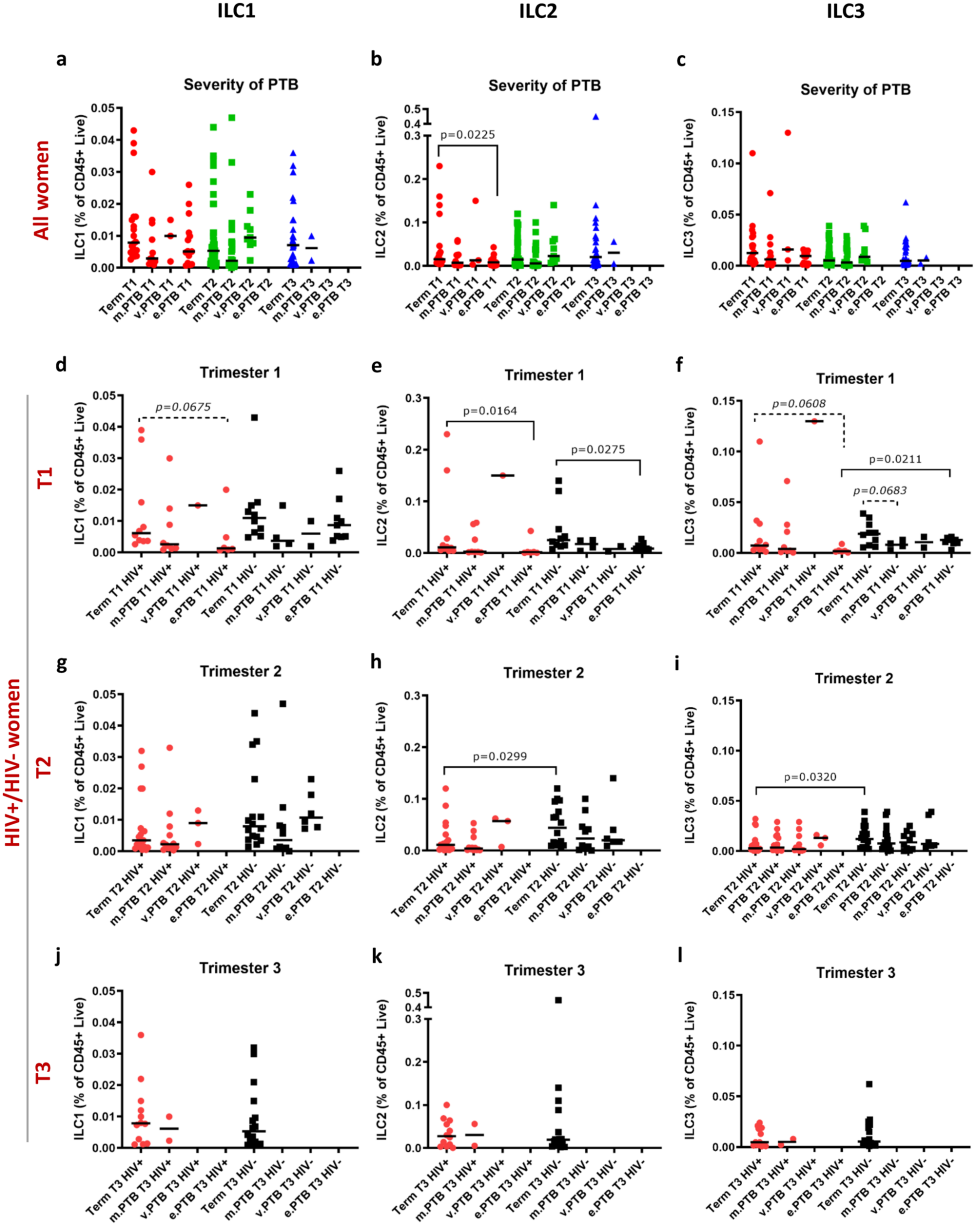
ILC1, ILC2 and ILC3 cells of HIV positive and/or HIV negative women with different severities of preterm birth or term birth. (**a**) ILC1, (**b**) ILC2 and (**c**) ILC3 cell frequencies of women who had moderate preterm (m.PTB), very preterm (v.PTB), or extreme preterm (e.PTB) births compared to those who delivered at term. (**d**–**f**) First (T1), (**g**–**i**) second (T2) and (**j**–**l**) third (T3) trimester ILC1, ILC2 and ILC3 frequencies, respectively, of HIV positive (HIV+) and HIV negative (HIV−) women who had term, moderate preterm, very preterm, or extreme preterm births. Data bars at median.

ILC frequencies were also analysed according to severity of PTB (Fig. 4a–c). In the first trimester, ILC2 cells were significantly lower in women who experienced extreme PTB compared to those with term births (Fig. 4b). When stratified according to HIV status, HIV+ women who would go on to have an extreme PTB had a significant reduction in first trimester ILC2 frequencies compared to mothers delivering at term, with a similar trend seen in ILC1 and ILC3 frequencies (Fig. 4d–f). A reduction in ILC2s was also seen in the first trimester of HIV− women with an extreme PTB compared to term birth (Fig. 4e). No second and third trimester data points were available for extreme PTB (Fig. 4g–l).

### ILCs are lower in mothers who deliver preterm after spontaneous onset of labour

**Figure 5:**
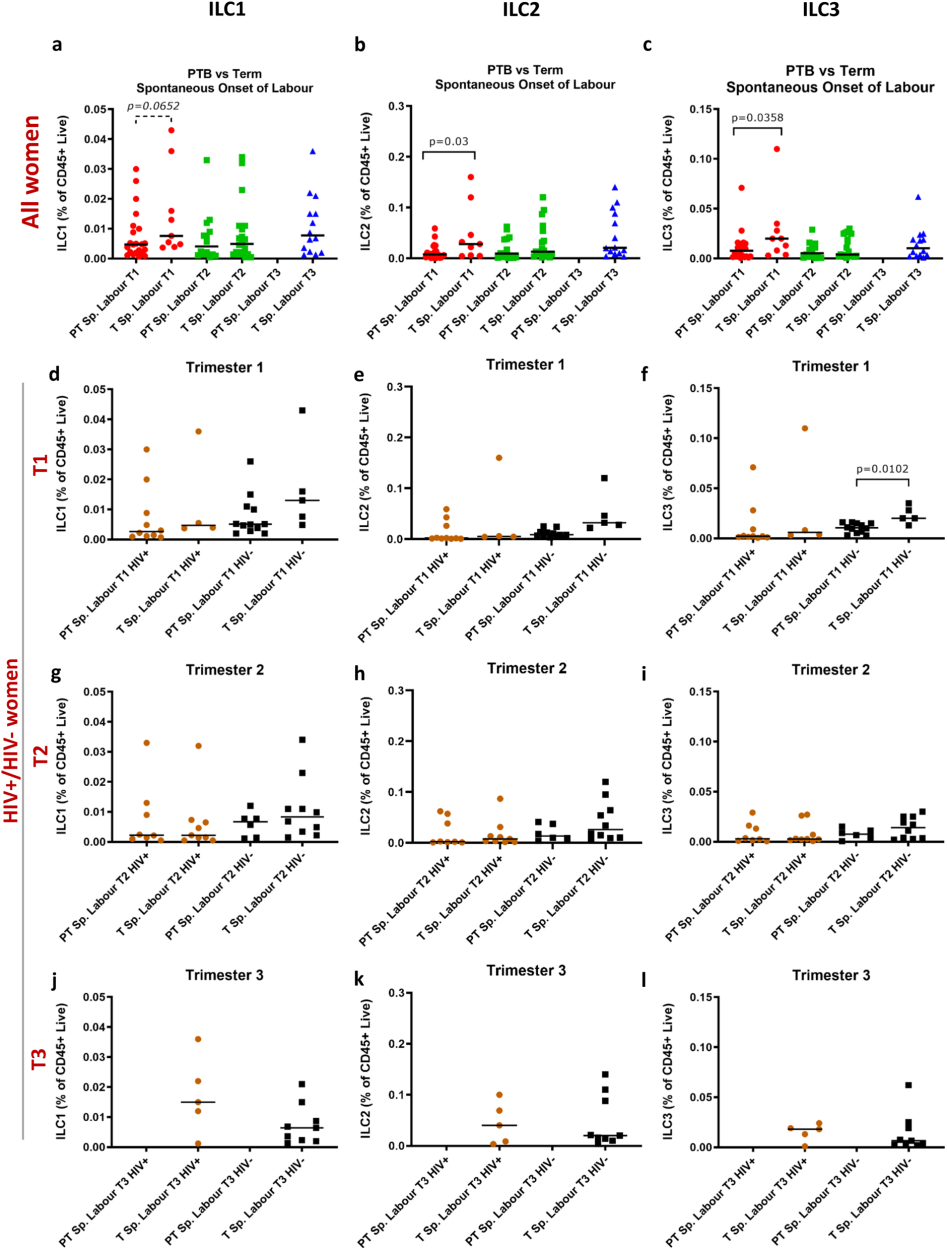
ILC1, ILC2 and ILC3 cells of HIV positive and/or HIV negative women with spontaneous onset of labour and preterm or term births. (**a**) ILC1, (**b**) ILC2 and (**c**) ILC3 cell frequencies of women with spontaneous onset of labour who delivered preterm (PT) compared to those who delivered at term (T). (**d**–**f**) First (T1), (**g**–**i**) second (T2) and (**j**–**l**) third (T3) trimester ILC1, ILC2 and ILC3 frequencies, respectively, of HIV positive (HIV+) and HIV negative (HIV−) women with spontaneous onset of labour who delivered preterm (PT) or at term (T). Data bars at median.

To investigate further the changes in ILC populations between mothers who delivered preterm or at term, we compared the ILC frequencies of those with spontaneous onset of labour within these two groups. During the first trimester of women with spontaneous onset of labour, ILC2 and ILC3 cells were significantly lower among women who delivered preterm compared to those who delivered at term, and the same trend was seen in ILC1s (*p* = 0.0652) (Fig. 5a–c). When stratified according to HIV-status, there was a stepwise decrease in ILC frequencies in women with spontaneous onset of labour in the first and second trimester, although the differences between HIV− women who delivered at term and HIV+ women who went on to deliver preterm were not significant (Fig. 5d–i). No data points were available for third trimester preterm spontaneous labour births (Fig. 5j–l).

Among women with spontaneous onset of labour ILC frequencies were also analysed according to severity of PTB (Supplementary Fig. S3). In the first trimester all three ILC subsets showed a trend for lower frequencies according to severity of PTB (Supplementary Fig. S3). Among HIV− women with spontaneous onset of labour, ILC2s and ILC3s were significantly reduced in the first trimester in those who experienced extreme and moderate PTB, respectively, compared to term birth, and a similar trend was seen for ILC1 (Supplementary Fig. S3).

## Discussion

To our knowledge, this is the first study to investigate the frequencies of all three ILC subsets in peripheral blood throughout pregnancy in HIV positive and HIV negative women. In line with previous reports in non-pregnant individuals^16,21,23,24^, we demonstrate that the frequencies of ILC1, ILC2 and ILC3 cells are reduced in pregnant HIV+ women compared to HIV− women. Although HIV+ women were older than HIV− women, there is no significant association between maternal age and ILC frequencies in our data set (data not shown). Furthermore, we identify the specific periods during which reduced ILC levels are observed: the first trimester for ILC3s with a trend for ILC1s and ILC2s, and the second trimester for all three ILC subsets. Our data indicate that HIV-associated ILC depletion prior to pregnancy, which likely occurs soon after acute HIV infection, is maintained during pregnancy.

Preterm birth is the leading cause of neonatal and child mortality and can result in lasting morbidities^1,2^. We show that preterm birth is associated with significantly lower levels of first trimester ILC1s and ILC2s. Moreover, those who experience extreme preterm births (< 28 weeks gestation) have significantly lower levels of ILC2s in the first trimester compared to term. HIV+ women who deliver preterm have the lowest ILC frequencies, whereas HIV− women with term births have the highest ILC frequencies; with significant differences between these two groups in the first trimester among all three ILC subsets and the second trimester for ILC2s. The stepwise decrease in ILCs from HIV− term births to HIV+ PTB, suggests that processes which occur during HIV infection and result in PTB may be linked by reduced ILC frequencies, which are lowest when PTB-related processes occur in conjunction with HIV infection. Importantly, in pregnancies resulting in spontaneous onset of labour, the first trimester difference in ILC frequencies between women who deliver preterm and at term is also seen, as well as the first and second trimester trend for the lowest and highest ILC frequencies to occur in HIV+ women with PTB and HIV− women with term births respectively. This indicates the differences in ILC levels predict spontaneous preterm labour and are not limited to births that are physician initiated due to other fetal or maternal conditions.

We had delivery and postnatal samples from a limited number of HIV+ women, allowing us to track changes in ILC frequencies throughout pregnancy and into a non-pregnant period. No changes occur in ILC frequencies over the course of pregnancy in HIV+ or HIV− women, and in HIV+ women there is no significant difference in ILC frequencies during pregnancy compared to delivery and 6 weeks postnatal. However, other studies report altered T cell dynamics during pregnancy, which remain stable during the pregnancy of HIV+ as well as HIV− women and then increase postpartum^36–39^. In our investigation, ILC1s and ILC2s appear to increase following pregnancy, but these differences are not significant, likely due to the small number of delivery and postnatal samples (n = 5 and 6, respectively). Also, pregnancy related immune modulations are known to continue into the postpartum period for up to a year^40–42^. Therefore, future investigations would benefit from an increase in delivery and postnatal sample sizes as well as comparable samples for HIV− and non-pregnant women.

Some reports suggest a link between the timing of ART initiation before or during pregnancy and the risk of an adverse pregnancy outcome, including preterm birth^7^. However, we find no difference in ILC frequencies between women who initiate ART preconception or post-conception, and ILCs are reduced in both groups compared to HIV− women. This lack of association may be due to the reported link between the timing of ART initiation and adverse pregnancy outcomes being based on selection bias, as previously noted^43^.

ILCs are present in the human endometrium as well as the decidua, suggesting their involvement in human pregnancy^25–30,44^. For instance, neutrophils participate in spiral artery remodelling, a process required for the provision of an adequate blood supply to the developing fetus^45^. ILC3s are implicated in this process by their location in the human decidua during the first trimester of pregnancy, where they reside in close proximity to decidual neutrophils and express GM-CSF and IL-8, which have been demonstrated to promote neutrophil migration and survival^27,46^. In addition, neutrophil expression of heparin-binding EGF-like growth factor and IL-1Ra, both factors involved in tissue remodelling and pregnancy maintenance, is induced by decidual ILC3-derived GM-CSF, and in instances of spontaneous miscarriage fewer neutrophils are found in the decidua basalis in the first trimester compared to healthy pregnancies^46^. During the first trimester, IL-17 and IL-22, signature cytokines of ILC3s, promote the survival, proliferation and invasion of human trophoblast cells, suggesting a supportive role of ILC3s in these early processes of pregnancy^47,48^. In contrast, during the later stages of pregnancy ILC3s are increased in the decidua parietalis of women who experience spontaneous preterm labour compared to term births^28^. ILC1s secrete IFN-γ as part of type 1 immune responses, but during murine pregnancy IFN-γ plays a vital role in the process of arterial remodelling^49^, furthermore its production by uterine ILC1s increases during murine gestation^25^. In humans IFN-γ is found in the endometrium where its functions include the inhibition of decidual renin^50^, an angiogenic factor involved in the intrauterine renin–angiotensin system vital for placental development and involved in spiral artery remodelling. Therefore, depletion of ILC1s and ILC3s in the early stages of pregnancy may contribute to impaired placentation and arterial remodelling resulting in adverse perinatal outcomes. A more severe depletion of ILCs would be expected to lead to a more severe phenotype, as is indeed observed for ILC levels in relation to severity of PTB in our study, especially in HIV+ women.

ILC2s release type-2 cytokines including IL-4, IL-5, and IL-13 in response to helminth infection, allergen and epithelial injury^18^. In murine pregnancy, ILC2s increase throughout gestation, along with IL-5 and IL-13 expressing ILCs, and are the most abundant uterine ILC subset^25,31^. In a mouse model of pregnancy loss, abortion mice display higher levels of uterine ILC3s but lower levels of uterine ILC1s and ILC2s accompanied by an increase in the serum levels of IFN-γ, IL-17A and IL-22, and a decrease in IL-5 and IL-13^34^. As well as mediating resistance to helminth infection, ILC2s promote tissue repair through the release of amphiregulin and IL-13^18^ and therefore may contribute to the maintenance of homeostasis in the decidua. ILC2s are increased in the decidua basalis of women with spontaneous preterm labour compared to women who deliver preterm without labour^28^ suggesting an increase in ILC2 related functions may contribute to pathology in these women. Taken together these studies suggest that the role of ILCs in pregnancy maintenance and outcome is a complex one, which depends not only on their abundance but also their temporal and spatial location and levels of cytokine expression. Imbalances in cytokine production are associated with adverse pregnancy outcomes such as preterm labour^51^, and dysregulation in ILC levels and cytokine production in women who experience preterm birth, particularly HIV+ women, may impact the function of ILCs particularly as they relate to the processes of trophoblast survival and invasion, and arterial remodelling. A deeper understanding of the mechanisms by which ILCs contribute to pregnancy maintenance is required before therapies aimed at ILC immune modulation can be implemented safely and with success. For instance, we have shown that peripheral ILC frequencies are decreased in HIV+ women and women who deliver preterm, therefore it could be suggested that therapies to boost ILC numbers may be beneficial. However, a decrease in peripheral ILC levels in women who deliver preterm could be due to increased recruitment to the decidua, thereby contributing to pathological processes.

In the course of ILC development, it is thought that both mature ILCs and ILC precursors leave the bone marrow, circulate in the blood, and enter tissue sites where precursor ILCs mature at local tissue sites^18^. In the decidua CD34+ progenitors have been identified which express the Id2 transcription factor required for ILC development^52^ suggesting uterine ILCs may be of decidual origin. However, these decidual CD34+ progenitors are committed towards the natural killer cell fate^53^. This suggests decidual ILCs originate from the blood and that their numbers in the decidua correlate with peripheral levels. For example, a study by Wu et al. examined decidual as well as peripheral blood Th17 cells and found that while the proportion of Th17 cells increases in the peripheral blood of pregnant women compared to non-pregnant women, during the first trimester Th17 cells are found in greater proportions in the decidua compared to the peripheral blood^47^. The authors also found that secretion of CCL2 by decidual stromal cells was responsible for recruiting peripheral Th17 cells into the decidua^47^. ILCs express a range of chemokine receptors that play a role in their localisation to sites including the skin and the gut^54^ and likely contribute to their recruitment to the decidua and depletion from the peripheral blood during pregnancy. For instance, all three ILC subsets can express CXCR6^55^,which along with its ligand CXCL16 secreted by trophoblast and decidual stromal cells, is involved in the process of decidualisation in human pregnancy^56^. In addition, CCL20, an antimicrobial and ligand for CCR6 which is expressed by ILC2s and ILC3s^57,58^, is secreted by rat uterine epithelial cells in response to pathogen-associated molecular patterns^59^ and has been implicated in defence against HIV-1 infection in the human female reproductive tract^60^. This suggests ILCs may be further recruited to the uterus in response to HIV infection and could contribute to the decrease in circulating ILCs we observe in pregnant HIV+ women compared to pregnant HIV− women.

ILCs are required for pregnancy maintenance but an imbalance, for example due to increased recruitment from the periphery, could contribute to pathological inflammatory processes. For example, Xu et al. find that ILC2s and ILC3s are increased in the decidua of women with spontaneous preterm labour^28^. ILC2s are known to contribute to chronic Th2 biased disorders such as Th2 asthma^61^ and may similarly be involved in chronic inflammation at the maternal–fetal interface during preterm labour. Our finding that peripheral ILCs show a trend for a stepwise decrease in frequency from HIV− women who deliver at term and are lowest in women with the most severe pathology, i.e. are both HIV positive and go on to experience preterm birth, suggests a potential reciprocal increase in inflammatory responses at the decidua which contribute to preterm birth.

Apoptosis has been proposed as a mechanism of ILC depletion in HIV+ patients due to the identification of pro-apoptotic gene signatures, responsiveness to apoptotic signals, activation of caspase-3 and increased annexin-V expression in ILCs in HIV+ patients^16,23,24^. ILCs are thought not to be susceptible to HIV infection, as they are reported not to express CD4 or contain detectable levels of viral RNA following infection^62^. However, CD4 expressing populations of ILC1s have recently been identified which are susceptible to productive HIV infection, and may represent an additional mechanism of HIV-associated ILC depletion^24,63^. However, in HIV+ patients we observe further reductions in ILC levels associated with preterm birth, the mechanisms for which are unknown.

In summary, our data indicate that a low frequency of peripheral ILCs in early pregnancy may contribute to processes that result in preterm birth in HIV+ and HIV− women. While significant differences in ILC frequencies are seen in the first and second trimesters but not the third, this could be due to the limited number of third trimester samples, due to delivery before the planned third trimester sample collection. Therefore, ILCs may be involved in processes throughout the course of pregnancy that implicate them in preterm birth^3^. It is unknown how effects downstream of reduced peripheral ILC frequencies lead to pathological pregnancy outcomes. However, inflammatory processes are involved in the initiation of both term and preterm labour^3^ and our study adds to the body of knowledge on the immune cells involved in these processes. Changes in the function as well as the frequency of regulatory T cell subsets have been associated with preterm labour and preeclampsia^64–67^. Similarly, functional readouts of ILCs during HIV infection and pregnancy will be required to further define the role of ILCs during these distinct immunological periods, as well as the implication of their loss and role in preterm birth. A fuller understanding of these mechanisms will aid in the development of preventative and therapeutic interventions to reduce the global burden of preterm birth. Finally, peripheral ILC frequencies may serve as early predictive biomarkers for women at risk of delivering preterm, particularly as they can be identified at early stages of pregnancy when interventions may be most effective, and from a site that is easy to access.

## Methods

### Patients

Blood samples were obtained from women enrolled in a prospective pregnancy cohort study at Chris Hani Baragwanath Academic Hospital (CHBAH), Soweto, South Africa^13^. Women included in the study were black South African, living in Soweto, aged 18 years or over, with a spontaneous conception resulting in a singleton pregnancy. Women with multiple pregnancies, a body mass index > 35 kg/m^2^ or an intellectual or physical disability, were excluded. All women (i.e. both HIV+ and HIV− women) had a first trimester dating ultrasound scan and HIV testing was routinely offered to those not known to be HIV positive at enrolment. Medical, obstetric and HIV/ART history were collected from medical records, antenatal cards and/or interviews, and perinatal outcomes of interest were recorded at delivery, as previously reported^13^.

### Outcome definitions

Preterm birth (PTB) was defined as birth from 16^+0^ to 36^+6^ weeks gestation. Women with moderate PTB (32^+0^–36^+6^ weeks), very PTB (28^+0^–31^+6^ weeks) or extreme PTB (< 28 weeks) were also analysed separately.

### Antiretroviral therapy initiation definitions

In HIV positive women, ART initiation was defined as preconception (Pre) if started before the date of the last menstrual period or post-conception (Post) if initiated after the last menstrual period date. Post-conception ART initiation occurred before the first trimester sample date for most HIV positive patients, except for one patient for whom ART was initiated after the first trimester sample.

### Sample collection and processing

Between 27 November 2013 and 20 October 2015, trained study nurses collected peripheral blood samples in each trimester from HIV positive and HIV negative pregnant women, and at delivery and 6 weeks postnatally for a subset of the HIV positive women. Samples were separated into plasma and peripheral blood mononuclear cells (PBMCs) by standard density gradient centrifugation. PBMCs were frozen in a solution of 50% (v/v) FCS, 10% (v/v) DMSO and R10 media. Plasma and PBMCs were initially stored at − 80 °C. Samples were then shipped to Oxford on dry ice where they were stored in liquid nitrogen.

### Flow cytometry

Patient samples were chosen for analysis based on pregnancy outcome, rather than to be representative of HIV positive or HIV negative women. Frozen PBMCs were thawed in a water bath at 37 °C. Each vial of thawed cells (~ 2.5 × 10^6^ cells) was added to 50 μl of DNAse I solution (1 mg/ml) and suspended in warm R10 media (37 °C). To identify live cells, cells were stained with the Zombie Aqua Fixable Viability Kit [BioLegend] according to the manufacturer's instructions. Lymphocytes were identified using the CD45 Pacific Blue (HI30) marker. To identify lineage negative and ILC populations, cells were stained for a panel of markers demonstrated by Kløverpris et al.^16^ to identify peripheral ILCs in their study of ILCs in HIV positive women. To identify lineage negative populations cells were stained for CD3 PerCP-Vio700 (REA613), CD11c PerCP-Vio700 (MJ4-27G12), CD14 PerCP-Vio700 (REA599), CD19 PerCP-Vio700 (REA675), CD34 PerCP-Vio700 (AC138), CD41 PerCP-Cy5.5 (HIP8), CD94 PerCP-Vio700 (REA113), CD303/BDCA2 PerCP-Vio700 (201A), FcεR1α PerCP-Vio700 (REA758), TCR-αβ PerCP-Vio700 (REA652), and TCR-γδ (REA591) PerCP-Vio700. To identify ILC populations, cells were stained for CD161 PE-Vio770 (REA631), CD294/CRTH2 PE (BM16), CD56/NKp44 VioBright-FITC (AF12-7H3), CD127 APC-Vio770 (REA614) and CD117 APC (REA787) [antibodies from Miltenyi Biotec, except for CD294/CRTH2, CD45, CD41 and CD303/BDCA2 from BioLegend]. Cells were incubated with the antibody cocktail in the dark at room temperature for 15 min, then washed once in ice-cold staining buffer (PBS with 10% FBS) and fixed in 200 μl of 2% paraformaldehyde. Fixed cells were resuspended in 300 μl ice-cold staining buffer prior to acquisition on a LSR II flow cytometer [Becton Dickinson]. Fluorescence compensation was set using OneComp eBeads [Thermo Fisher Scientific], MACS Comp Bead Kit, anti-REA [Miltenyi Biotec], and a single stain of PBMCs using Zombie Aqua Fixable Viability Kit [BioLegend]. Flow cytometric analysis, including compensation for spectral overlap, was done using FlowJo V10 software [FlowJo LLC].

### Statistical analysis

Patient characteristics were analysed for normality and compared using the appropriate statistical tests (Mann–Whitney-U test or unpaired t test for continuous variables; Fisher’s exact test or Chi squared test for categorical variables). The Mann–Whitney U-test was used to compare median values between two groups within a trimester. The Kruskal–Wallis test was used to compare median values of more than two unpaired groups followed by Dunn’s multiple comparisons test to determine differences between two groups. *P* values < 0.05 were considered statistically significant. Above the graphs, solid or dashed horizontal brackets indicate statistically significant differences and those nearing statistical significance, respectively.

### Ethical approval

Written informed consent was obtained from all study participants upon enrolment. Ethical approval was obtained from the University of Oxford Tropical Research Ethics Committee (OxTREC) and the Human Research Ethics Committee (Medical) of the University of Witwatersrand, Johannesberg, South Africa. All experiments were performed in accordance with relevant guidelines and regulations.

## Supplementary information

Supplementary figures.
