# Supplementary material for: Innate lymphoid cells are reduced in pregnant HIV positive women and are associated with preterm birth
Source: Sci Rep. 2020 Aug 6;10:13265. doi: 10.1038/s41598-020-69966-0 (PMC7413261; doi:10.1038/s41598-020-69966-0)
Supplement: Supplementary file 1 — Supplementary figures. [file 41598_2020_69966_MOESM1_ESM.pdf]

# Innate Lymphoid Cells Are Reduced in Pregnant HIV Positive Women and Are Associated with Preterm Birth

Running title: Reduced ILCs link maternal HIV infection and preterm birth

**Charlene Akoto[a1], Christina Y. S. Chan[a1], Chrystelle O. O. Tshivuila-Matala[a, b], Krithi Ravi[a], Wei Zhang[a], Manu Vatish[a], Shane A. Norris[a, b], Joris Hemelaar[a, b *]**

[a] Nuffield Department of Women's & Reproductive Health, University of Oxford, The Women's Centre, John Radcliffe Hospital, Oxford, United Kingdom.

[b] South African Medical Research Council Developmental Pathways for Health Research Unit, Department of Paediatrics, School of Clinical Medicine, University of the Witwatersrand, Johannesburg, South Africa.

[1] These authors contributed equally to this work.

***Correspondence:**

Dr Joris Hemelaar

Nuffield Department of Women's & Reproductive Health, University of Oxford, The Women's Centre, John Radcliffe Hospital, Oxford, OX3 9DU, United Kingdom.

Email: joris.hemelaar@wrh.ox.ac.uk

# Supplementary Figures

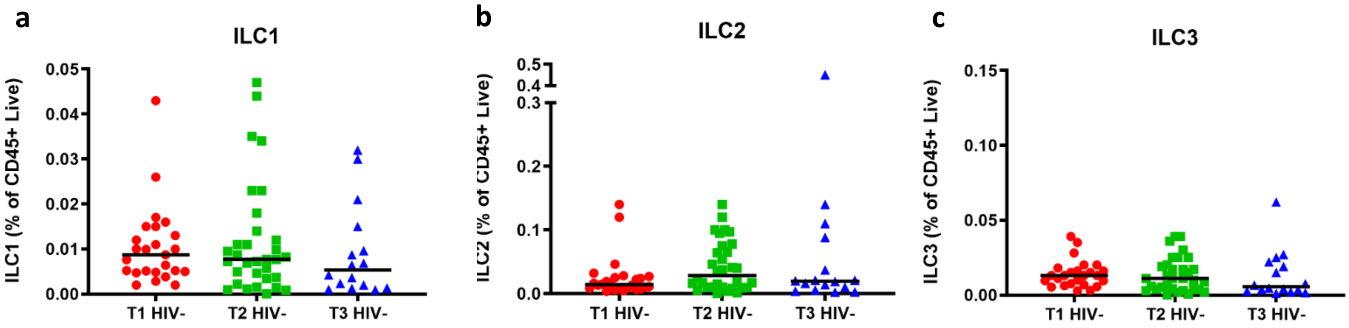


**Supplementary Figure S1. ILC1, ILC2 and ILC3 cells throughout the pregnancy of HIV negative women. (a)** ILC1, **(b)** ILC2 and **(c)** ILC3 cell frequencies of HIV negative (HIV-) women during the first (T1), second (T2) and third (T3) trimester.

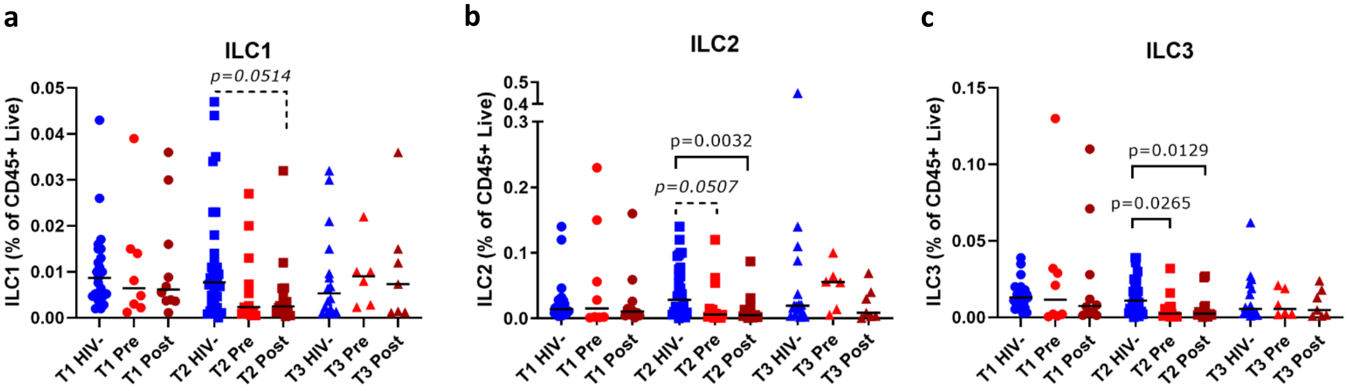


**Supplementary Figure S2. ILC1, ILC2 and ILC3 cells throughout the pregnancy of HIV positive women with preconception or post-conception antiretroviral therapy initiation compared to HIV negative women. (a)** ILC1, **(b)** ILC2 and **(c)** ILC3 cell frequencies during the first (T1), second (T2) and third (T3) trimester in HIV negative (HIV-) women or HIV positive women receiving preconception (Pre) or post-conception (Post) antiretroviral therapy.

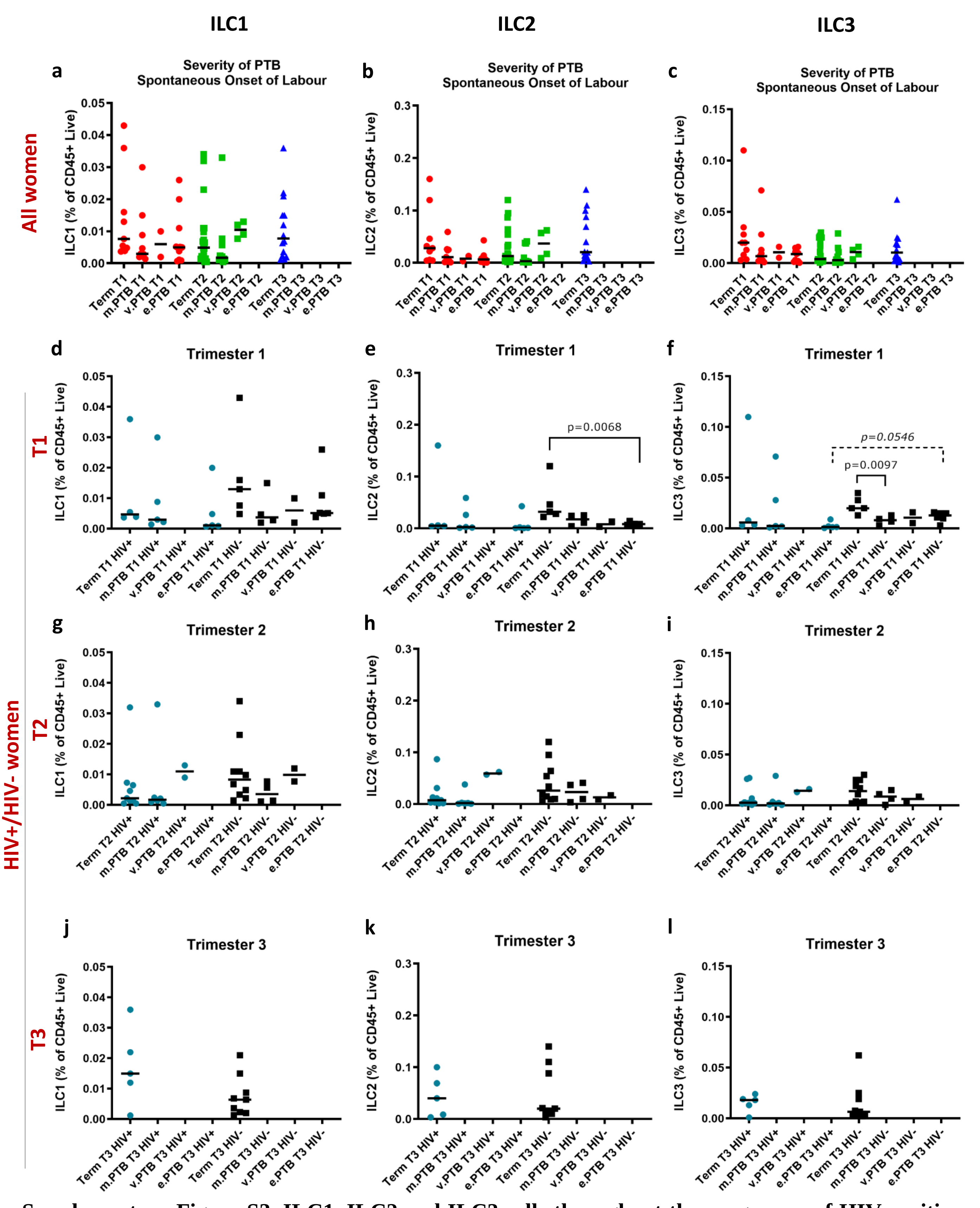


**Supplementary Figure S3. ILC1, ILC2 and ILC3 cells throughout the pregnancy of HIV positive and/or HIV negative women with spontaneous onset of labour and different severities of preterm birth or term birth.** **(a)** ILC1, **(b)** ILC2 and **(c)** ILC3 cell frequencies of all women, both HIV positive and HIV negative, with spontaneous onset of labour who had preterm (PTB), moderately preterm (m.PTB), very preterm (v.PTB), or extreme preterm (e.PTB) births compared to those who delivered at term. **(d-f)** First (T1), **(g-i)** second (T2) and **(j-l)** third (T3) trimester ILC1, ILC2 and ILC3 frequencies, respectively, of HIV positive (HIV+) and HIV negative (HIV-) women with spontaneous onset of labour who had term, preterm, moderately preterm, very preterm, or extreme preterm births.
